# Editorial: Immunology of Psoriatic Disease

**DOI:** 10.3389/fimmu.2019.00657

**Published:** 2019-03-29

**Authors:** Eva Reali, Nicolò Costantino Brembilla

**Affiliations:** ^1^Department of Life Sciences and Biotechnology, University of Ferrara, Ferrara, Italy; ^2^Laboratory of Translational Immunology, I.R.C.C.S. Istituto Ortopedico Galeazzi, Milan, Italy; ^3^Department of Pathology and Immunology, Faculty of Medicine, University of Geneva, Geneva, Switzerland

**Keywords:** psoriasis, psoriatic arthritis, metabolic syndrome, cardiovascular comorbidities, autoimmune/autoinflammatory disease, inflammation, skin immunopathology, cytokines

Psoriasis is a chronic recurrent T cell-mediated inflammatory skin disease with a strong genetic predisposition. The disease is associated with joint manifestations (psoriatic arthritis, PsA), developing in about 30% of patients, and with comorbidities such as metabolic syndrome.

Genome-wide scans provided the first insight into the pathogenesis of psoriasis and showed that the HLA-C^*^06:02 allele in the psoriasis susceptibility locus 1 on chromosome 6 accounts for up to 50% of disease heritability. Other gene variants associated with psoriasis are involved in the IL-23/IL-17 axis, CD8^+^ T cells differentiation, antigen processing, NF-κB/IL-1/TNF axis, and type-I interferon response (1).

The picture that emerges is that psoriasis is a complex disease with autoimmune and autoinflammatory components that involves the interplay between keratinocytes, microvascular endothelium, dendritic cells (DCs), and T cells, generating a self-sustaining inflammatory cycle around the TNF/IL-23/IL-17 axis. Epidermal CD8^+^ T lymphocytes producing IFNγ and IL-17 may represent a major autoimmune mechanism in disease pathogenesis (2, 3).

Many questions remain unanswered and new scenarios open up based on the increasing evidence that has been recently provided. The mechanisms leading to the initial manifestations of psoriasis remain uncertain, and the exact characterization of the autoimmune and autoinflammatory responses occurring in psoriasis patients, as well as the mechanisms linking skin with extra-cutaneous and systemic manifestations await clarification.

In this Research Topic, we invited scientists to summarize the latest advances on immunological mechanisms of psoriatic disease. The topic starts with three manuscripts exploring the possible autoimmune nature of psoriasis and its extra-cutaneous manifestations. Prinz reviews the refined approach that led to the characterization of autoreactive CD8^+^ T cells specific for melanocyte-derived ADAMTLS5 antigens presented by HLA-C^*^06:02, strengthening the evidence of an autoimmune component in psoriasis pathogenesis. HLA-C appears central to the promotion of organ-specific T cell responses due to its ability to present positively charged skin self-antigens.

Then, Frasca et al. describe their original research on the characterization of autoantibodies to neutrophil LL-37 antimicrobial peptide in patients presenting PsA. The authors show that autoantibodies to LL-37 are elevated in PsA synovial fluid and correlate with clinical inflammatory markers. Although antibody formation may represent a secondary effect of the priming of LL37-specific T helper cells, these findings provide new insights in the autoimmune aspects linking psoriasis and its extra-skin manifestations.

In the next article, De Jesús-Gil et al. overview the translational studies showing microbial trigger of skin-homing CLA^+^ cells. The authors provide evidence that the CLA^+^ fraction of circulating T cells secrete IL-17A, IL-17F, and IL-9 when stimulated with *Streptococcus pyogenes*. In addition, they report that CLA^+^ T cells in psoriasis patients respond to skin *S. pyogenes* and *C. albicans* extracts, suggesting a relationship between memory T cells and environmental microbes. Finally, the authors underline how CLA^+^ T cells activated by streptococcal antigens in tonsils could migrate to the skin where they recognize keratin-derived self-antigens presenting homology with streptococcal proteins.

In this context, Casciano et al. outline the conceivable sequence of T cell-mediated events of the psoriatic inflammatory cascade. These include an initial phase in which autoreactive CD8^+^ T cells could play a major role, a second phase involving both self-reactive and polyclonal CD4^+^ T amplified by the IL-23/IL-17A axis, and an antigen-independent downstream recruitment of circulating CXCR3^+^ T cells. In this view the egress of T cells from the skin and their recirculation through the blood could represent a link between cutaneous psoriasis and its systemic and joint manifestations.

The topic proceeds with four articles addressing the complex cytokine and cellular networks in psoriasis. Schön and Erpenbeck uncover the importance of the IL-23/Th17 axis in the cross-talk between innate and adaptive immune responses, including new players such as IL-17-producing innate lymphoid cells and unconventional γδT cells. The authors further analyze the T cell-neutrophil-keratinocyte loop leading to the amplification of the immune responses, particularly referring to the role of neutrophil extracellular traps. These latter may modulate the immune system by reducing the activation threshold of T cells and by favoring the presentation of neutrophil autoantigens (e.g., antimicrobial peptides) to antigen presenting cells (APCs).

Further exploring the cytokine network in psoriasis, Brembilla et al. review the presence and function of cytokines belonging to the IL-17 family, focusing on the role of isoforms other than IL-17A. The functions of IL-17F and IL-17C are highlighted, as well as a recently discovered role for IL-17E (IL-25). This latter, over-produced by keratinocyte in psoriasis, promotes the activation of macrophages, leading to the amplification of the psoriatic inflammation and the recruitment of innate immune cells, such as neutrophils, to the skin.

With reference to other keratinocyte-derived cytokines, Bridgewood et al. report a role for IL-36γ in the induction of IL-23 and TNFα by macrophages, as well as in endothelial activation and angiogenesis.

The topic continues with the manuscript from Albanesi et al., highlighting the role of keratinocytes in the establishment and amplification of the psoriatic inflammation. In response to a trigger, keratinocytes participate to the early phase of disease by releasing β-defensins, S100 proteins and antimicrobial peptides that in turn activate APCs to initiate an adaptive response. Epidermal cells may also be a source of autoantigens such as neolipids recognized by CD1a-restricted T cells and keratins cross-recognized by streptococcal-specific T cells. Once activated, keratinocytes function as amplifier of the inflammation by producing chemokines and cytokines, particularly those belonging to the IL-1 family, such as IL-36γ (see articles from Schön and Erpenbeck; Bridgewood et al.)

The next three manuscripts describe novel disease patho-mechanisms. Dolcino et al. investigate the long non-coding RNA (lncRNA) signature of PsA patients. lncRNAs control gene expression at multiple levels and are emerging as new players in autoimmune disease. The authors integrated this information with gene expression and microRNA data from the same cohort, using protein-protein interaction network and pathway enrichment analysis. They identify a restricted set of lncRNAs modulated in PsA that target genes highly expressed in the disease, such as genes involved in inflammatory response and bone remodeling. These lncRNAs were in addition involved in the modulation of lipid metabolism and mTOR pathways, suggesting their link to disease comorbidity.

Next, Chimenti et al. review the role of oxidative stress in psoriasis and PsA. A focus is given to mediators released from mast cells and mitochondrial activity (e.g., tryptase and cytochrome c), and their role as amplifiers of the inflammatory loop.

Finally, Mylonas and Conrad provide an interpretation of paradoxical psoriasis, an immunologically different form of psoriasis induced in some patient following anti-TNF treatment. In classical psoriasis, the initial release of IFNα activates myeloid DCs to produce TNFα and IL-23. TNFα subsequently maturates DCs and limits IFNα production via a negative feedback loop, leading to activation of IL-23-dependent T cell responses. In patients developing paradoxical psoriasis, the therapeutic blockade of TNF inhibits DC maturation, leading to a sustained IFNα production and the occurrence of an inflammation independent of T cell.

The three articles that follow discuss the association of psoriasis with cardiovascular comorbidity.

First, Boehncke travels through the cellular and molecular events at the basis of this association: genetic predisposition, skin inflammation, shared pathogenetic mechanisms. While all these aspects are likely involved, none explains why psoriasis might actually be regarded as an independent risk factor for cardiovascular diseases. The framework of the “psoriasis march” represents the possible missing link: psoriasis is a systemic inflammatory condition leading to insulin resistance and endothelial dysfunction, resulting in increased vascular stiffness and atherosclerosis.

Then, Sajja et al. provide a detailed view of the immunological mechanisms shared by psoriasis and atherogenesis. Activation of Th1 and dysfunction of Treg cells emerge as a main feature. Additional data sustain a role for innate immune cells, in particular the low-density granulocytes (LDGs). These cells have an enhanced capacity to from extracellular traps, which represents a source of autoantigens on one hand, and a direct cause of endothelial injury and cardiovascular plaque rupture on the other hand.

Therapies for psoriasis targeting these shared mechanisms may potentially prevent the rate of cardiovascular events. Treatments aimed at reducing inflammation, such as methotrexate and IL-1β neutralization, provided a proof of this concept. In this context, Caiazzo et al. show that none of the recently-licensed biologics (e.g., anti-IL-12/23 and anti-IL-17 agents) significantly impacts the cardiovascular risk, despite the analysis is biased by a short follow-up period. Overall, these reviews underline that an intimate link actually exists between psoriasis and cardiovascular diseases. This justifies a comprehensive approach to the management of psoriasis, including proper advice and a regular screening and monitoring for cardiovascular factors.

The next three manuscripts explore novel therapeutic avenues. As of now, inhibition of IL-17A proved to be the most effective approach, demonstrating the key role of Th17 cells in clinical settings. Tang et al. embrace the rationale of inhibiting the development of Th17 cells (with logic similar to that behind the use of anti-IL-23 agents), rather than neutralizing IL-17A. This could be more beneficial since it would result in the inhibition of multiple pro-inflammatory cytokines. The originality of their vision relies on the use of small molecule antagonist targeting RORγt, the master regulator of Th17 cells.

Besides targeting Th17 cells, the possibility of restoring the functionality of Treg cells for therapeutic purpose may represent an attractive idea. Chen et al. report that the beneficial effect of PSORI-CM02 formula, a traditional Chinese medication based on the combination of five herbs, relies on the amplification of Treg in disfavor of Th17 cells.

In the last article of the collection, Buerger approaches the question of the therapeutic intervention from a different angle, and drives the reader into the intracellular epidermal processes induced by the immunological network. Focus of the review is the role of the mTORC1 cascade as regulator of the epidermal homeostasis, independently of its known function as immune-modulator. While active during cellular proliferation, keratinocytes need to switch off the mTORC1 pathway to proceed into terminal differentiation and cornification, partially via the activation of an autophagy response. Cytokines such as IL-17A and TNF aberrantly activate mTORC1 resulting in hyperproliferation and the arrest of the differentiation process. In this respect, mTOR could be seen as a novel therapeutic target for psoriasis.

In conclusion, this collection well proves how our comprehension of the patho-physiology of psoriasis has radically changed in the last decade. Constantly more gaps are getting filled, and multiple novel mechanistic insights added to our previous knowledge, spanning from the autoimmune nature of the disease to the possible causes underlying extra-cutaneous manifestations and comorbidity. A hard work awaits however scientists and clinicians in the future, as patient deserve increasingly more specific and efficacious therapeutic or even curative options.

## Author Contributions

All authors listed have made a substantial, direct and intellectual contribution to the work, and approved it for publication.

### Conflict of Interest Statement

The authors declare that the research was conducted in the absence of any commercial or financial relationships that could be construed as a potential conflict of interest.

## References

[1] Harden JL Krueger JG Bowcock AM. The immunogenetics of Psoriasis: a comprehensive review. J Autoimmun. (2015) 64:66–73. 10.1016/j.jaut.2015.07.008 26215033 PMC4628849

[2] Boehncke WH Schon MP. Psoriasis. Lancet. (2015) 386:983–94. 10.1016/S0140-6736(14)61909-7 26025581

[3] Gallais Serezal I Classon C Cheuk S Barrientos-Somarribas M Wadman E Martini E. Resident T cells in resolved psoriasis steer tissue responses that stratify clinical outcome. J Invest Dermatol. (2018) 138:1754–63. 10.1016/j.jid.2018.02.030 29510191

